# Hyperhidrosis: an update on prevalence and severity in the United States

**DOI:** 10.1007/s00403-016-1697-9

**Published:** 2016-10-15

**Authors:** James Doolittle, Patricia Walker, Thomas Mills, Jane Thurston

**Affiliations:** 1 Brickell Biotech, 2600 SW 3rd Avenue #300, Miami, FL 33129 USA; 2 Burke Inc., 500 West 7th Street, Cincinnati, OH 45203 USA

**Keywords:** Hyperhidrosis, Excessive sweating, Prevalence, Severity, Impact

## Abstract

Current published estimates of the prevalence of hyperhidrosis in the United States are outdated and underestimate the true prevalence of the condition. The objectives of this study are to provide an updated estimate of the prevalence of hyperhidrosis in the US population and to further assess the severity and impact of sweating on those affected by the condition. For the purposes of obtaining prevalence, a nationally representative sample of 8160 individuals were selected using an online panel, and information as to whether or not they experience hyperhidrosis was obtained. The 393 individuals (210 female, 244 non-Hispanic white, 27 black, mean age 40.3, SE 0.64) who indicated that they have hyperhidrosis were asked further questions, including body areas impacted, severity of symptoms, age of onset, and socioemotional impact of the condition. Current results estimate the prevalence of hyperhidrosis at 4.8 %, which represents approximately 15.3 million people in the United States. Of these, 70 % report severe excessive sweating in at least one body area. In spite of this, only 51 % have discussed their excessive sweating with a healthcare professional. The main reasons are a belief that hyperhidrosis is not a medical condition and that no treatment options exist. The current study’s findings with regard to age of onset and prevalence by body area generally align with the previous research. However, current findings suggest that the severity and prevalence are both higher than previously thought, indicating a need for greater awareness of the condition and its associated treatment options among medical professionals.

## Introduction

Hyperhidrosis is a skin disorder characterized by sweating in excess of what is required to maintain regulation of normal body temperature [15]. Hyperhidrosis is classified as either primary or secondary in nature. Primary hyperhidrosis is idiopathic; it results from over-activity of the sympathetic nerves and involves a limited body area, most often the axillae (underarms), palms, soles, or craniofacial regions [21]. Secondary hyperhidrosis results from an underlying medical condition or use of prescription medications and implicates the entire body [21].

Hyperhidrosis can range in severity from mild dampness to severe dripping and can result in substantial impairment in quality of life. This includes limitations in work and social relationships, physical and leisure activities, and impairments in emotional and mental health [1, 2, 4, 12, 17, 20]. The negative impact caused by excessive sweating has been reported to be similar to, if not greater than, the negative impact caused by conditions, such as psoriasis and other chronic diseases [5, 17].

Given the widespread negative impact and potential severity of hyperhidrosis, it is important to understand how many people (in the United States) are afflicted with the disorder. The most commonly cited estimate of prevalence of primary hyperhidrosis in the United States is based on a study published in 2004 [23]. Based on a survey mailed to 150,000 households in 2002, this study estimates that 2.8 % of the U.S. population has primary hyperhidrosis. They report prevalence rates are highest among those 25–64 years old (3.5–4.5 %) and lowest among those younger than 12 years old (0.5–0.7 %). Furthermore, they estimate that just over half (50.8 %; prevalence rate 1.4 %) of those with hyperhidrosis experience excessive sweating of the underarms (i.e., axillary hyperhidrosis) either in isolation or in combination with another body area, making it the most commonly affected area.

Prevalence estimates of primary (focal) hyperhidrosis outside of the United States are all higher and vary widely, ranging from 4.6 % in Germany, 5.5 % in Sweden, 12.3 % in Vancouver, Canada, 12.8 % in Japan, and 14.5 % in Shanghai, China [1, 7, 16, 20]. Prevalence estimates that include both primary and secondary hyperhidrosis range from 13.9 % in Japan, 16.3 % in Germany, 16.7 % in Vancouver, 18.4 % in Shanghai, and 20.3 % in Sweden [1, 7, 16, 20].

Modalities to treat hyperhidrosis, namely axillary hyperhidrosis, include topical (e.g., topical aluminum chloride hexahydrate), oral (e.g., anticholinergics), injectable (e.g., botulinum toxin type A), iontophoresis (i.e. mild electrical current through water), microwave thermolysis (e.g., miraDry), and surgical (e.g., endoscopic thoracic sympathectomy), and all vary in their efficacy (in terms of reduction in severity and duration of effect), side effects, ease of use, and cost [6, 9–11, 17, 22]. Topical treatments, such as prescription strength aluminum chloride antiperspirants, are the recommended first-line treatment option for most cases of hyperhidrosis [21]. However, while they can be moderately effective in the short term, they have shown less satisfactory results in the long run and are oftentimes associated with intolerable skin irritation at the application site. More efficacious treatment options have the primary drawbacks of being relatively invasive, costly and/or have less compelling evidence regarding their safety [21]. Additional treatment methods that are under development include: new versions of laser therapy, micro-focused ultrasound treatment, topical botulinum toxin type A, and topical anticholinergics [3, 8, 13, 18].

Given higher prevalence rates in other countries, we hypothesize that the prevalence of hyperhidrosis in the United States is conservative. Furthermore, the actual prevalence is likely higher than the previous estimates suggest, because hyperhidrosis is both underreported by patients and underdiagnosed by healthcare professionals (HCPs) [1, 19]. The primary purpose of the present study is to provide an updated estimate of hyperhidrosis prevalence overall and by body area among the US population, and to assess the severity and associated impact of sweating on those affected by hyperhidrosis.

## Materials and methods

### Prevalence estimate sample

A total of 275,904 invitations were sent via email for a self-administered online survey. A total of 12,363 people entered the survey (response rate 4.5 %), with 8160 people providing information as to whether or not they experience hyperhidrosis. The sample sources were two online panel providers, which both consist of nationally representative samples of U.S. households. The current study’s sample was balanced to represent US census parameters (Table 1).

**Table 1:** Sample characteristics for prevalence estimate

	No. of individuals	Sample used for prevalence estimate (%)	Census (%)^a^
All	8160	100	
Gender × age			
Male (under 40 years old)	1332	35.3	37
Male (40+ years old)	2440	64.7	63
Female (under 40 years old)	1579	36.0	34
Female (40+ years old)	2809	64.0	66
Race/ethnicity			
Non-Hispanic White or Euro-American	5178	63.4	61
Black, Afro-Caribbean or African American	759	9.3	13
Hispanic/Latino	1002	12.3	17
East Asian/Asian American	432	5.3	6
Other (including Indian American, Native American/Alaskan Native, and Middle Eastern/Arab American)	789	9.7	3
Household income			
Under $25k	1436	17.6	25
$25–50k	2318	28.4	26
$50–75k	1775	21.8	19
$75–100k	1125	13.8	12
$100–150k	1023	12.5	12

^a^Customized census reporting obtained from Marketing Systems Group (MSG) based on 2014 Current Population Survey (CPS) data

The numbers in Table 1 reflect the characteristics of the adults who responded to the survey. Any parents were also asked how many children (regardless of age) they have, if any, who experience hyperhidrosis or excessive sweating. Children under the age of 18 reported as having hyperhidrosis here were included in the prevalence estimate.

### Hyperhidrosis sufferers

A subset of the participants identified as having hyperhidrosis for the prevalence estimate were asked more specific questions about their experiences with hyperhidrosis. A total of 393 (210 female, 244 non-Hispanic white, 27 black, mean age 40.3, SE 0.64) participants completed the more detailed survey (305 adults answering for themselves and 88 parents answering on behalf of their child with hyperhidrosis). In addition to the two online panel providers mentioned previously, the International Hyperhidrosis Society (IHS) was enlisted to help recruit parents of children with the condition. Comparisons of ratings between parents of children obtained from the online panels and from the IHS were compared and no meaningful differences in responses were evidenced.

### Survey description

The survey used in this study included questions designed to identify individuals with hyperhidrosis, irrespective of whether they have received a medical diagnosis. For the purposes of this study, hyperhidrosis was defined as

A condition that involves chronic excessive sweating of the underarms, hands, feet, face, groin, or other bodily areas, which is much more than what is normal, and occurs regardless of temperature, exercise or situation, and may have an impact on quality of life.

Female participants who experienced both hyperhidrosis and night sweats/hot flashes that may be associated with menopause were disqualified if they self-reported the inability to distinguish between the two conditions.

Other information collected in the survey included age of onset, occurrence, and severity of symptoms (severity was assessed by body area using the four-point, single-item Hyperhidrosis Disease Severity Scale (HDSS) [14]. The HDSS asks respondents to rate the severity of their symptoms based on tolerability and interference with daily activities. Specifically, respondents are asked whether their sweating is:

1. never noticeable and never interferences with daily activities;
2. tolerable but sometimes interferes with daily activities;
3. barely tolerable and frequently interferes with daily activities or;
4. intolerable and always interferes with daily activities.

Any body area where the sweating was classified as never noticeable/never interferes was not included in the calculation for occurrence of symptoms by body area.

## Results

### Prevalence

The prevalence of hyperhidrosis in the survey sample was 4.8 %, which represents approximately 15.3 million people when extrapolated to the US population. The prevalence rate is highest among 18–39 years old (8.8 %) and lowest among adults 65+ years old and children/adolescents (2.1 %; Table 2).

**Table 2:** Prevalence of hyperhidrosis by age

	No. of individuals	US prevalence rate (%)
All	15,348,587	4.8
Age group (years old)		
<18	1,550,640	2.1
18+	13,797,947	5.6
18–39	8,401,202	8.8
40–64	4,409,345	4.2
65+	987,401	2.1

Interestingly, this study found that only 51 % of hyperhidrosis sufferers have discussed their condition with an HCP. Children/adolescents (<21 years old) with the condition are almost twice as likely to be seen by an HCP (81 %) than their adult counterparts (42 %; Table 3). The most commonly cited reasons for not discussing their condition with an HCP are that they do not think it is a medical condition (60 %), or that they believe that there is nothing that can be done to treat their excessive sweating (47 %). Of those who do see an HCP about their excessive sweating, only 53 % are diagnosed (73 % of children/adolescents, 43 % of adults; Table 3).

**Table 3:** Proportion of individuals with hyperhidrosis seeking treatment and getting diagnosed by age and severity

	Proportion (%) who discussed sweating with a healthcare professional	Proportion (% of total) diagnosed with hyperhidrosis
All	51	27
Age group (years old)		
<21^a^	81	59
21+	42	18
Severity		
HDSS score of 3 or 4 for at least one body area	52	31
HDSS score of 1 or 2 for all body areas	48	18

^a^Results based on parents’ responses

### Occurrence, severity, and age of onset by body area

Overall, 20 % of hyperhidrosis sufferers report that their excessive sweating is isolated to one body area. The most commonly reported body area where hyperhidrosis occurs is the underarms (Table 4). Sixty-five percent of respondents report experiencing axillary hyperhidrosis, either in isolation (10 % of total respondents) or in combination with other body areas (55 % of total respondents). The most commonly cited area that co-occurs with the underarms is the head/face (29 % of total respondents).

**Table 4:** Occurrence, severity, and age of onset by body area

	Occurrence (%)	Severity (% 3 or 4 on HDSS scale)	Mean age of onset (years) (SE)
Total	100	70	19.7 (0.5)
Underarms (axillary)	65	52	19.0 (0.6)
Head/face (craniofacial)	42	50	20.9 (0.8)
Hands (palmar)	40	54	16.6 (0.6)
Feet (plantar)	38	47	17.6 (0.7)
Under breasts	29	31	20.7 (0.9)
Back	28	39	20.2 (0.9)
Chest	27	39	21.1 (1.0)

Overall, 70 % report that their excessive sweating is severe in at least one body area. Severity in total and by individual body areas can be found in Table 4. Severe sweating rates range from 31 % under the breasts to 54 % for palmar hyperhidrosis.

The mean age of onset overall is just under 20 years (*M* 19.7, SE 0.5) and is fairly consistent across all body areas (Table 4), except that age of onset tends to be younger for those with palmar (*M* 16.6, SE 0.6) and plantar (*M* 17.6, SE 0.7) hyperhidrosis.

### Impact of hyperhidrosis

Hyperhidrosis sufferers feel a great negative impact on their lives due to the condition. About three quarters of respondents report that excessive sweating has had at least some negative impact on their social life, sense of wellbeing, emotional health, and mental health (Table 5).

**Table 5:** Proportion of individuals with hyperhidrosis who report a minor or major negative impact of hyperhidrosis in various areas

	Social life (%)	Sense of well-being (%)	Emotional health (%)	Mental health (%)	Physical health (%)	In sports/exercise (%)	Sexual health (%)	Work relationships (%)
Major + minor neg. impact^a^	77	76	75	72	60	57	54	52
Major neg. impact	30	27	32	29	18	23	14	16
Minor neg. impact	47	49	43	43	42	34	40	36

^a^Sorted left to right based on % major + minor negative impact

Overall, a large majority of respondents agree that excessive sweating is embarrassing (85 %), and has caused them to experience anxiety (71 %; Table 6). Furthermore, 35 % agree that they have had to sacrifice many important things in their life because of the condition. Given the widespread negative impact of hyperhidrosis, it is not surprising that over half (54 %) of respondents say that they would pay anything for a treatment to stop their excessive sweating.

**Table 6:** Proportion of individuals with hyperhidrosis who agree with the following statements in total and by severity

	Total (% agree^a^)	HDSS score of 3 or 4 for at least one body area (% agree)	HDSS score of 1 or 2 across all body areas (% agree)
Sweating excessively is very embarrassing	85	90	73
My excessive sweating has definitely made me experience anxiety	71	79	51
I really hate doing all the things that I must do to deal with my excessive sweating	60	69	37
I would pay anything for a treatment that would stop my excessive sweating	54	61	37
I feel angry that I need to deal with sweating when others do not	53	61	34
I feel very alone in suffering from excessive sweating	46	54	27
My excessive sweating has made me depressed	36	43	20
I have had to sacrifice many important things in my life because of my sweating	35	43	16

^a^Percent of respondents providing a rating of 4 or 5 on a five-point Likert scale where 1 = “strongly disagree” and 5 = “strongly agree”

## Discussion

This study seeks to update the current understanding of the prevalence of hyperhidrosis in the United States and its clinical presentation in terms of age of onset, body areas, and severity. The current study’s findings with regard to age of onset and prevalence by body area generally align with the previous research [10, 23]. However, findings from this study suggest that the severity and prevalence (in the United States) are both higher than previously thought.

Many studies have demonstrated that hyperhidrosis can have a severe and pervasive impact on quality of life [2, 4, 5, 12, 17, 23]. In terms of HDSS categories, the previous studies have estimated that approximately one-third of people with hyperhidrosis present with a severe form of the disease [23]. This study estimates that 70 % of people with hyperhidrosis experience severe sweating on at least one body area, as indicated by the sweating being either barely tolerable or intolerable and frequently or always interfering with daily activities. This study also finds further evidence that quality of life is significantly impaired for hyperhidrosis sufferers. About three quarters of respondents report that excessive sweating has had at least some negative impact on their social life, sense of wellbeing, emotional health, and mental health, and more than half are desperate enough to indicate that they would pay anything for a treatment to stop the sweating.

The most comprehensive review of prevalence of hyperhidrosis in the United States was provided by Strutton and colleagues [23]. Using a paper survey mailed to 150,000 households, they estimated that 2.8 % (7.8 million individuals) are affected by hyperhidrosis. They concluded that the “disease affects a much larger proportion of individuals in the United States than previously thought” (p. 247). Current findings, given all of the methodological details discussed herein, indicate that the prevalence of hyperhidrosis is about 2 % higher than previously thought and currently impacts about 15.3 million individuals, or 4.8 % of the U.S. population. Of these 15.3 million individuals, only just over half will discuss their excessive sweating with an HCP and, ultimately, only 27 % will be diagnosed. There is further evidence, therefore, that the condition remains both underreported and underdiagnosed. The most often-cited reasons for not seeking treatment are beliefs that hyperhidrosis is not a medical condition and that there is nothing that can be done to treat their excessive sweating.

Even though the 4.8 % estimate is higher than previously thought for the U.S. population, it is still far below prevalence reported in other countries [1, 7, 16, 20], indicating that the current finding may still be a conservative estimate. The reasons for differences in prevalence estimates are not known, although demographical, geographical, diagnostic criteria, and methodologies used for estimating prevalence differ between studies [16]. This study may also differ from other reports due to some limitations.

This study shares a limitation with Strutton and colleagues in that survey research is, by its nature, subject to selection bias. While the online panel partners leveraged for this study are comprised of a nationally representative set of potential respondents, online research can only reach individuals who have access to online resources. However, an advantage of using an online panel relative to an omnibus survey mailer (method used by Strutton and colleagues) is that the incoming sample can be continually monitored and adjusted. Furthermore, outgoing invitations were balanced to compensate for known response bias. These methods resulted in a sample which accurately represents the US population.

Another potential limitation of the current research is that the identification of people with hyperhidrosis is not based on a medical diagnosis or clinical assessment. Instead, it is self-reported based on a clinical definition of the disease that was provided to the respondents. However, given that so many patients never see an HCP (current study estimates 49 %) and ultimately remain undiagnosed (current study estimates 73 %), self-assessment will continue to be necessary to better estimate the true prevalence. That said, menopausal females who self-reported an inability to differentiate between night sweats/hot flashes and the symptoms of hyperhidrosis were excluded from the study, but other clinical assessments or diagnostic criteria may have excluded additional individuals. This was also a limitation of the Strutton and colleagues study, so the comparison of prevalence estimates between the two studies is not hindered. However, the exclusion of menopausal females who cannot differentiate between their symptoms may be a contributor to the current study’s prevalence being lower than what is reported in other countries.

## Conclusions

The hyperhidrosis prevalence estimates provided by this research indicate that hyperhidrosis impacts an additional 2 % of the United States population above what has previously been estimated (15.3 million people or 4.8 %) [23]. Of these people, only about half discuss their condition with an HCP suggesting that the actual prevalence may be, in fact, underreported. For individuals who seek treatment, their physicians will need more effective (first-line) treatment options at their disposal to have more confidence in identifying and diagnosing the condition. Among the majority of those who do not seek treatment, there is a lack of awareness of hyperhidrosis as a medical condition that has treatment options available. The fact that only half of those with hyperhidrosis do not consult an HCP is especially alarming given that current results indicate 70 % of individuals experience severe sweating in at least one area. Furthermore, the widespread negative impact of hyperhidrosis, especially on sufferers’ social lives, sense of wellbeing, and emotional and mental health, is further documented by these individuals. Ultimately, there are 15.3 million people in the United States (far more than previously thought) with hyperhidrosis that can result in severe impairment in their daily lives, and many of them are not diagnosed or treated.
